# The presence of microplastics in commercial salts from different countries

**DOI:** 10.1038/srep46173

**Published:** 2017-04-06

**Authors:** Ali Karami, Abolfazl Golieskardi, Cheng Keong Choo, Vincent Larat, Tamara S. Galloway, Babak Salamatinia

**Affiliations:** 1 Laboratory of Aquatic Toxicology, Department of Environmental and Occupational Health, Faculty of Medicine and Health Sciences, Universiti Putra Malaysia, 43400 Selangor, Malaysia; 2 Discipline of Chemical Engineering, School of Engineering, Monash University Malaysia, 47500 Selangor, Malaysia; 3 HORIBA Jobin Yvon S.A.S., 231, rue de Lille – 59650, Villeneuve d’Ascq, France; 4 College of Life and Environmental Sciences, University of Exeter, Exeter, EX4 4QD, Devon, United Kingdom

## Abstract

The occurrence of microplastics (MPs) in saltwater bodies is relatively well studied, but nothing is known about their presence in most of the commercial salts that are widely consumed by humans across the globe. Here, we extracted MP-like particles larger than 149 μm from 17 salt brands originating from 8 different countries followed by the identification of their polymer composition using micro-Raman spectroscopy. Microplastics were absent in one brand while others contained between 1 to 10 MPs/Kg of salt. Out of the 72 extracted particles, 41.6% were plastic polymers, 23.6% were pigments, 5.50% were amorphous carbon, and 29.1% remained unidentified. The particle size (mean ± SD) was 515 ± 171 μm. The most common plastic polymers were polypropylene (40.0%) and polyethylene (33.3%). Fragments were the primary form of MPs (63.8%) followed by filaments (25.6%) and films (10.6%). According to our results, the low level of anthropogenic particles intake from the salts (maximum 37 particles per individual per annum) warrants negligible health impacts. However, to better understand the health risks associated with salt consumption, further development in extraction protocols are needed to isolate anthropogenic particles smaller than 149 μm.

Since their mass production in the 1950s, global plastic production has been increasing, which exceeded 322 million tons in 2015^1^. Mismanaged plastic waste could find their ways to oceans^2^. Meanwhile, continuous fragmentation of large plastic objects has resulted in the accumulation of smaller particles called microplastics (MPs, sized between 1 and 1000 μm^3^,^4^). Also, MPs may be directly introduced to the aquatic environments through their primary sources (e.g., synthetic sandblasting media, cosmetic formulations, textiles)^5^,^6^,^7^. The widespread distribution of MPs in aquatic bodies is well documented, such as in the Celtic sea^8^, Laurentian Great Lakes^9^, Persian Gulf^10^, and in sub-tropical gyres^11^. Accordingly, it is expected that products originating from the contaminated water bodies are also loaded with MPs.

Several studies have shown the presence of MPs in seafood products like clams^12^ and fish^13^. Therefore, the consumption of seafood products could be a significant route of exposure to MPs in humans. For instance, top European shellfish consumers are expected to ingest up to 11,000 plastic particles per annum^14^. Microplastics might be of health concern since they have been shown to carry hazardous chemicals^15^,^16^ and microorganisms^17^.

Despite the relatively well-documented occurrence of MPs in seafood products, little is known about MP loads in abiotic saltwater products, which are expected to inevitably contain contaminants from the water. Sodium is an essential element for the human body to maintain homeostasis and is mainly consumed as common salt (sodium chloride, NaCl)^18^. Commercial sea and lake salts are mainly produced through a crystallization process as a result of seawater evaporation or naturally occurring brine under the combined effects of sunlight heat and wind^19^,^20^. There are concerns over the potential transfer of water contaminants into sea salt after the crystallization and concentration process^20^. In the only available study on abiotic products, Yang *et al*.^21^ showed the presence of up to 681 MPs/kg in salts originating in China. However, the chemical composition of individual MP particles was not determined, with MP identification based instead on grouping the particles according to their morphological features and analyzing representative samples through Fourier Transform Infrared Spectroscopy (FT-IR). Nevertheless, visual sorting is not the most reliable method to identify MPs^22^,^23^ as this could significantly overestimate the concentration of anthropogenic materials. No report is available, however, on MP loads in salt samples from other regions of the world that are potentially consumed by around 6 billion people, excluding billions of other organisms such as cattle that need to consume salt on a regular basis^24^. In this study, we further investigated the presence of MPs by extracting particles from 17 different brands of salt originating from 8 countries over 4 continents. Density separation and visual identification were employed to initially isolate MP-like particles. Finally, all particles were analyzed by micro-Raman spectroscopy for their chemical composition.

## Results

**Figure 1:**
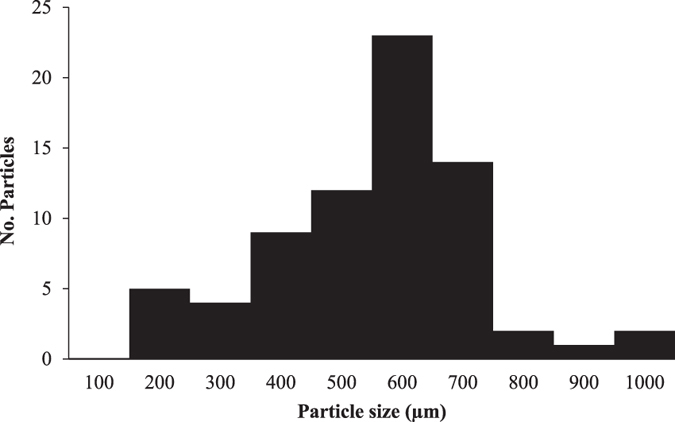
Histogram of the number of isolated particles across different sizes.

**Figure 2:**
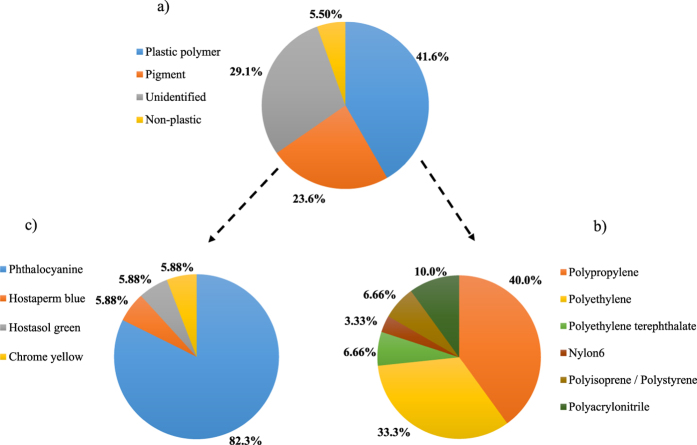
Chemical composition of the isolated particles. (**a**) Pie chart of the chemical composition of the isolated particles from all salt samples and the corresponding proportion of different (**b**) plastic polymers and (**c**) pigments.

**Figure 3:**
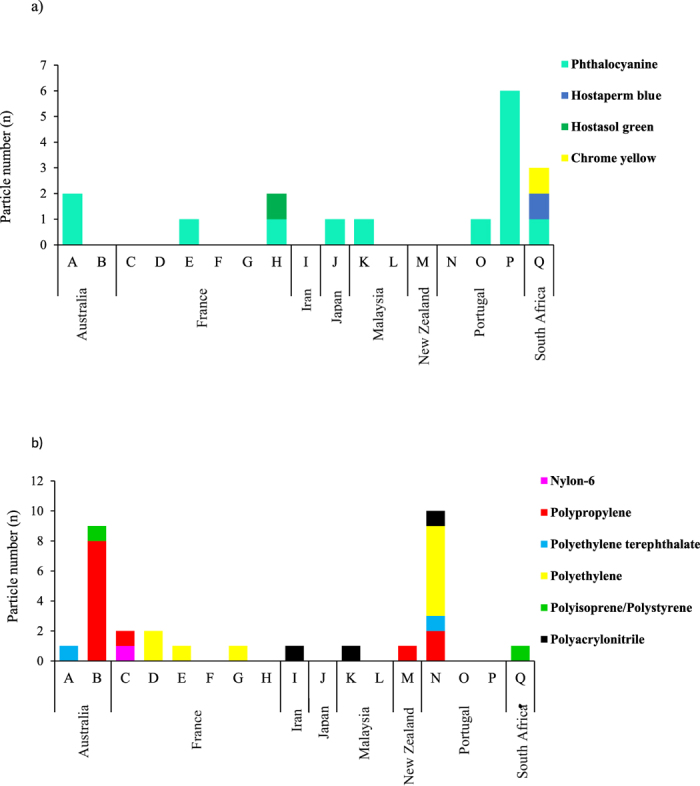
Stacked bar charts of the isolated particles across the salt brands. Stacked bar chart of the number of (**a**) plastic polymer and (**b**) pigment particles isolated from different salt brands.

The presence of a large quantity of white sediment in the lake salt from Malaysia (Table 1) blocked the 149 μm-pore size filter membrane. These particles were identified as calcium carbonate (CaCO_3_) using Raman spectroscopy. Therefore, this sample was excluded from MP analysis. A total of 72 MP-like particles were isolated from 16 salt brands. The average particle size (mean ± SD) was 515 ± 171 μm. The size of the smallest particle was 160 μm and the largest sized 980 μm. Figure 1 presents a histogram of the number of the particles sorted by size. As depicted by Fig. 2a, 30 particles (41.6%) were confirmed as plastic polymers, 17 particles (23.6%) were pigments, 21 particles (29.1%) were not identified, and 4 particles (5.50%) were non-plastic items (i.e., amorphous carbon). The major plastic polymers were PP at 40.0% of the total plastic polymers followed by PE (33.3%), polyethylene terephthalate (PET; 6.66%), polyisoprene/polystyrene (6.66%), polyacrylonitrile (10.0%), and polyamide-6 (nylon-6, NY6; 3.33%) (Fig. 2b). Particles identified as pigments were phthalocyanine (82.3%), chrome yellow (5.88%), hostasol green (5.88%), and hostaperm blue (5.88%) (Fig. 2c). The abundance of MPs per salt sample ranged from 0 per kg in the salt sample # France-F (i.e. Country of origin: France, brand F) to 10 in the salt sample # Portugal-N. Figure 3a and b are the stacked bar chart of the number of plastic polymer and pigment particles isolated from each salt brands, respectively.

**Figure 4:**
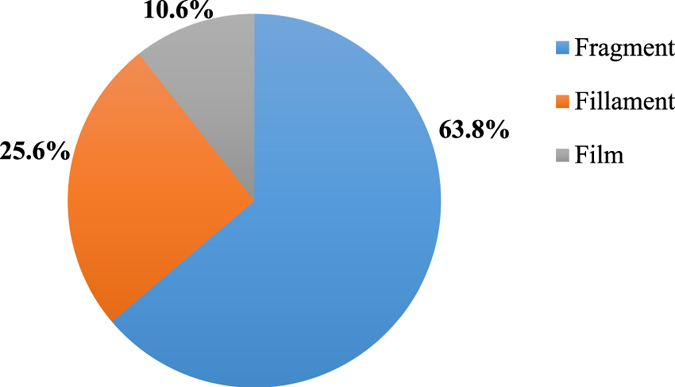
Pie chart of microplastic type.

**Figure 5:**
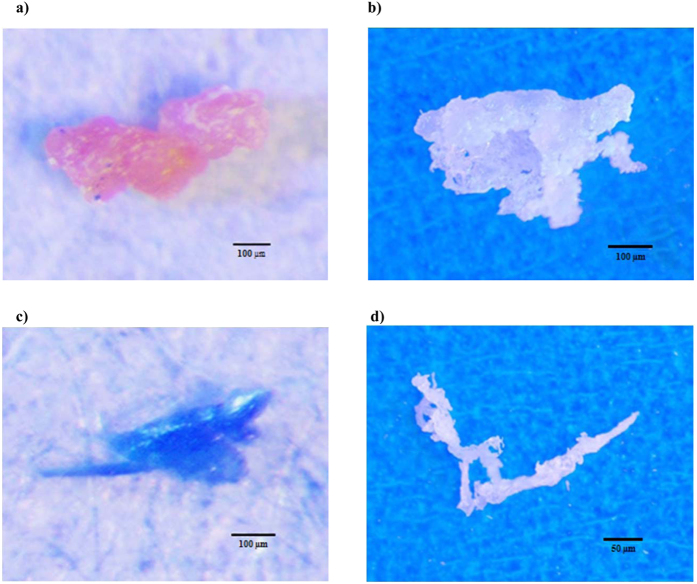
Microscopic images of some of the extracted particles. A (**a**) polyisoprene/polystyrene, (**b**) polyethylene, and (**c**) pigment (phthalocyanine) fragment. Image d is a nylon-6 filament.

With regards to the particle morphology, the predominant type were fragments (63.8%) followed by filaments (25.6%), and films (10.6%) (Fig. 4). No sphere beads were isolated from the salt samples. Figure 5 illustrates the microscopic images of some of the isolated particles. Supplementary Information Fig. 1a–e present the microscopic images and spectra from some of the isolated MPs along with the spectra of reference materials.

## Discussion

In this study, we developed a simple and cost-effective protocol to isolate MPs from salt samples. The presence of insoluble particles quickly blocked the filter papers with pore sizes of 2.7, 8, and 22 μm. Initially, we hypothesized that the blockage was due to the presence of organic materials. However, lack of changes on the filtration after KOH digestion showed the presence of digestion-resistant organic or inorganic materials. Therefore, dilutions with deionized water, followed by filtration through a membrane with a larger pore size (149 μm), was the simplest and quickest method to isolate MPs from the salt samples. Our recent study demonstrated a high efficcincy of 4.4 M NaI solution to separate (recovery rate >95%) plastic polymers from high-density particles like shell fragments from bivalves and sand grains^3^. To minimize the chance of overestimating or underestimating MP prevalence in the salt samples, in addition to NaI extraction, we implemented microscopical examination and Raman spectroscopy. Despite processing the samples through NaI extraction and visual identification, 5.50% of the particles were amorphous carbon (Supplementary Information Figure 1e), which underscores the necessity of using spectroscopic techniques to identify the chemical composition of the isolated particles. Micro-Raman spectroscopy is a highly specific technique used to identify the composition of biological, mineral or polymer samples. It offers a number of advantages such as analysis of microscopic particles while being non-invasive towards the samples^23^,^25^. In addition, Raman measurements do not depend on the transmission of light through the particle, which consequently allows an accurate analysis of thicker or pigmented particles^23^. Some of the earlier studies solely relied on the morphological characteristics of MPs, like shape and color to identify MPs in environmental samples^26^,^27^ while others partially confirmed the particle composition through a random selection of the isolated particles^21^,^28^. Although observation is an indispensable part of polymer identification, it cannot be employed as a stand-alone technique for particle characterization because it is unlikely to be sufficient to identify the polymer type through morphological features.

Consistent with the findings of this study, fragments and filaments have been reported as the main form of MPs^29^,^30^. The absence of microbeads in the salt samples may indicate their low prevalence in aquatic environments. Polypropylene (PP) and polyethylene (PE) were the most abundant plastic polymers in the salt samples (40.0% and 33.3%, respectively) which is consistent with reports on their wide distribution in the marine environment^22^. The presence of these polymers in salt samples could be due to the low density of PP (0.90–0.91 g/cm^3^) and PE (0.91–0.96 g/cm^3^) allowing these to float on the water surface and be readily directed into saltpans. In addition, their low density may facilitates their spread by becoming airborne. Polyisoprene/polystyrene (styrene-isoprene-styrene block copolymer) were the other detected synthetic polymers in the salt samples. These polymers are used when elasticity and easy processing is required, such as in adhesives and sealants^31^. A few isolated particles from the salt samples had a similar composition to their packaging. This might indicate degradation of the packaging materials leading to the contamination of the salt product. Nevertheless, this hypothesis was rejected since all the fragments or films were highly corroded, indicating their long-term presence in the environment.

Almost one fourth of the isolated particles were identified as pigments (phthalocyanine, chromate yellow, and hostaperm blue) because the strong Raman signal of these pigments hindered the identification of plastic polymers. Phthalocyanine is a synthetic pigment and is extensively used in the plastics industry^32^ and was the main pigment isolated from the salt samples (Fig. 2c). Hostaperm blue falls under the copper phthalocyanine chemical class and is an industrial dye that is mainly used in the plastics industry^32^. Victoria blue is commonly used as a coloring agent in polyacrylic fibers (Supplementary Information Figure 1a)^30^ that are mainly introduced to the marine environment through the washing of clothes after passing through sewage treatment plants^34^. Meanwhile, lead chromate (yellow) pigment is a toxic compound that has extensive applications in paints and plastic industries owing to its excellent light-fastness and low cost^33^,^35^. Earlier studies have attributed exposure to lead chromate pigment with incidents of bronchial carcinoma^36^, cerebrovascular disease^37^, and nephritis^38^ in humans. However, the occurrence of only one particle of lead chromate pigment per Kg of salt # South Africa-Q, poses a negligible threat to the health of consumers.

Initially, we hypothesized that the pigment particles might be paint particles. However, since none of the extracted particles shared similar mechanical properties as paint particles like brittleness^39^, we suggest the absence of paint particle in the salt samples. Other than plastics, pigments are widely used in other materials like textile, rubber, and fiberglass^40^,^41^. Van Cauwenberghe *et al*. suspected that the particles identified as copper phthalocyanine, polychloro copper phthalocyanine, and permanent red in deep-sea sediments^42^, as well as copper phthalocyanines and haematite in bivalves^14^ to be plastic materials. Similarly, in this study we could confirm that the pigmented particles had an anthropogenic origin but could not ensure they were MPs.

In the present study, a significant portion (29.1%) of the particles was not identified by Raman spectroscopy. Photo-degradation and weathering are the two major factors suggested as the causes for variation in the spectroscopic spectra of polymers such as PVC^23^. Moreover, the presence of additives could alter the polymer spectra and hinder comparisons with the reference library^23^,^42^. Another reason for having unidentified samples is the lack of a comprehensive spectra library to identify mixed samples^23^.

Lake salt from Malaysia was excluded from MP analysis because this contained a large volume of sediments, which were later identified as CaCO_3_. Calcareous sedimentation is a common process occurring in lakes mainly due to the assimilation of carbon dioxide by photosynthesizing plants and/or seasonal temperature effects on the solubility of carbon dioxide and calcite^43^. It should be noted that the other lake salt (# Iran-I) did not contain calcareous sedimentation, which shows the variation in calcium contents among different lake salt brands.

In 2010, the global daily sodium consumption was 3.95 g/day (equivalent to 9.88–10.2 g salt/day^44^) corresponding to 3.6 to 3.7 Kg salt per annum. The number of anthropogenic particles (MPs and pigments) detected in the salt samples ranged from 0 (sample # France-F) to 10 (sample # Portugal-N) MPs/Kg. Based on this data, humans could ingest a maximum of 37 plastic particles annually. This should take into account that this maximum value is based on the assumption that sea salt is the sole source of sodium intake. Other sources of sodium supply are food additives like monosodium glutamate and preservatives. Therefore, in real scenarios, the maximum MPs intake is probably even less than 37 particles.

Microplastics may cause adverse effects to organisms through causing micro injuries (mainly in the case of fragments)^4^ or the release of pollutants that had been sorbed during their prolonged incubation in the water. In the case of the latter, MPs have shown the ability to sorb persistent organic pollutants (POPs)^45^ and subsequently desorb them under simulated gut conditions^46^. However, recent studies have argued the lower importance of MPs as a vector for translocation of POPs to aquatic biota as compared to other routes like food and water^15^,^16^. Despite a potentially high concentration of POPs and other contaminants in MPs, the combination of their small particle size and low prevalence indicate that the consumption of sea salt does not appear to be a major route for the contaminant transfer into the human body as compared to other sources like water and food. Due to technical limitations, however, we could only quantify the level of particles larger than 149 μm. The prevalence of smaller particles in the salt samples, however, might be higher than the larger ones. Smaller sizes could facilitate their translocation into other organs and, therefore, cause a higher degree of toxicity. For example, in a study by Lu *et al*.^47^, 20 μm polystyrene (PS) microspheres accumulated in the gills and gut of zebrafish (*Danio rerio*), while 5 μm microbeads were incorporated into the gills and gut as well as liver. Further advances in isolation techniques are needed to quantify smaller MP particles before making a more accurate justification on the health impacts of sea salt consumption. However, it should be taken into account that salt is not the only edible item that has been shown to contain MPs. These have been previously detected in clams^12^, mussels^14^, fish^13^ and unexpectedly in honey^48^ as well as beer^49^. Therefore, the long-term consumption of various products containing MPs might become a concern.

Due to their low density and slow degradation, plastics are becoming the chief cross-border contaminant that often travels far from their original source. Hence, MPs found in the salt samples of one country could have been produced by another country thousands of miles away. A potential solution to this global dilemma requires a strong commitment from all the countries to make a substantial improvement in plastic disposal and recycling.

## Conclusions

The results of this study did not show a significant load of MPs larger than 149 μm in salts originating from 8 different countries and, therefore, negligible health risks associated with the consumption of salts. The increasing trend of plastic use and disposal^50^, however, might lead to the gradual accumulation of MPs in the oceans and lakes and, therefore, in products from the aquatic environments. This should necessitate the regular quantification and characterization of MPs in various sea products.

## Methods

### Materials

A total of 17 brands of salt from Australia, France, Iran, Japan, Malaysia, New Zealand, Portugal, and South Africa were purchased from a Malaysian market. NaCl (analytical grade) was purchased from Merck (Darmstadt, Germany), and sodium iodide (NaI), potassium hydroxide (KOH), and ethanol 95% were supplied by R&M Chemicals (UK). Solutions of NaI (4.4 M) and KOH (10% w/v) were prepared by dissolving the powder/pellet in ultrapure deionized water. GF/D microfiber filter paper (pore size 2.7 μm), and filter membrane No. 540 and 541 filter membranes (hardened ashless, pore size 8 and 22 μm, respectively) were purchased from Whatman. The 149 μm filter membranes were supplied by Spectrum Laboratories (USA). High-density polyethylene (HDPE), low-density polyethylene (LDPE), PP, PS, PET, polyvinyl chloride (PVC), NY6, and nylon-66 (nylon-66, NY66) virgin plastic fragments were supplied by Toxemerge Pty Ltd (Australia). About 10% of particles were sized below (D10) 40 μm, 50% below (D50) 140 μm, and 90% (D90) below 310 μm.

### Extraction method development

In an attempt to employ a solvent to dissolve the salt particles or efficiently digest the organic materials, we compared digestion using a KOH solution^3^ with appropriate dilutions with deionized water. A 20 g sample of sea salt from sample # Australia-A was placed into a 250 mL DURAN glass bottle (Schott, Germany) sealed with a premium cap and pouring ring (Schott, Germany), and then 200 mL KOH solution or deionized water were added (1:10 w/v). The bottle was manually shaken until the full dissolution of salt. These solutions were then incubated at 40 °C for 48 h and were vacuum filtered through 2.7, 8, or 22 μm membranes. Results showed that both solvents failed to pass through the 2.7, 8 or 22 μm filter membranes owing to the presence insoluble or digestion resistant materials. Therefore, dilution with deionized water was used to dissolve salts due to its cost effectiveness, accessibility, and inertness to the plastic polymers. Next, another experiment was run to find a filter membrane with the most suitable pore size.

### Filtration optimization

Upon poor filtration of the salt solution (dissolved in water or digested with KOH solution) through a 22 μm filter membrane, a series of sieves were individually used to identify the best pore size for filtration. Briefly, a solution of sea salt was prepared in deionized water (1:10 w/v) and added into different 8-inch testing sieves-full height stainless steel frame and wire (pores sizes 38, 45, 53, 63, 90, 106, 125, or 150 μm; Daigger Co., Vernon Hills, IL, USA) and mechanically shook on an orbital shaker at 200 rpm for 2 h. The salt solution could only fully pass through the 150 μm pore size sieve. Therefore, disposable filter membranes with the nearest pore size to 150 μm were chosen (Spectrum Laboratories, USA; Pores size: 149 μm) for assessing MP loads in the salt samples.

### Method validation

To validate the dilution method, 5 g of NaCl (analytical grade), 0.05 g of crushed shell from Asian green mussel (*Perna viridis*), 0.05 g of sand grains, and 0.1 g of HDPE, LDPE, PP, PS, PET, PVC, NY6 or NY66 were weighed on a scale (0.1 mg precision) and then added into a 100 mL laboratory bottle in triplicate. The bottles were filled with 50 mL deionized water and shook manually until the complete dissolution of salt. After that, they were vacuum filtered through 8 μm-pore size filter membrane using a vacuum pump (Gast vacuum pump, DOA-P504-BN, USA) connected to a filter funnel manifold (Pall Corporation, USA). To separate the high-density particles (shell fragments and sand grains), the filter membrane was soaked in 10–15 mL of NaI (4.4 M, density: 1.5 g/cm^3^) in a 50 mL glass bottle and sonicated at 50 Hz for 5 min followed by agitation on an orbital shaker (200 rpm) for 5 min. Finally, the solution was centrifuged at 500 × g for 1 min, and the supernatant containing MPs was vacuum filtered through another 8 μm filter membrane. To ensure the full isolation of MPs, this stage was repeated again. Finally, the filter membrane was placed into a clean glass Petri dish and dried at 50 °C in an oven for 5 h.

**Figure 6:**
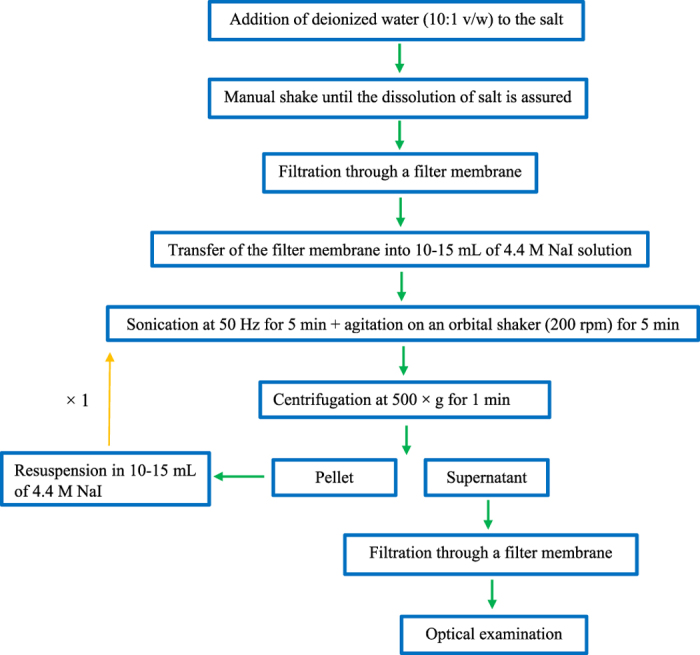
Flow diagram of the developed microplastic extraction protocol from salt samples.

No significant change was noticed in the weight of the filter membrane belonging to the procedural blank after filtration (Student’s t-test, p > 0.05) showing the full dissolution of NaCl. The recovery rates of all polymers were > 95%. Therefore, dissolution in deionized water followed by density separation using NaI was used to isolate MPs from the commercial salt samples (Fig. 6).

### Extraction and characterization of MPs in the commercial salt samples

#### Extraction

One package of sea salt (200–400 g) was mixed with 2–4 L of deionized water in a 2 or 5 L laboratory bottle and then vacuum filtered through a filter membrane (pore size 149 μm) to collect the insoluble materials. Between 3 and 5 salt packages (replicate) per brand were used to make a final weight of 1 kg. The filter membrane was placed into a 50 mL laboratory bottle and was subjected to NaI treatment as described earlier. The pellet was re-suspended in 10–15 mL of NaI, sonicated, agitated, and centrifuged to ensure the complete separation of MPs trapped within the insoluble materials.

### Characterization

#### Visual selection of the extracted particles

The filter membranes were inspected using a Motic SMZ-140 stereomicroscope (Motic, China). Visual inspection was performed and MP-like particles were sampled based on their morphological characteristics like color and shape. However, low-density particles such as carbon-based materials, plant tissues, and remains of invertebrate exoskeletons could not be excluded through density separation. The remains of these invertebrates were mostly yellow, light brown, or black in color, and possessed a soft texture and an even surface with smooth edges. The plant-based materials were dark brown or black. Extra care was taken when sampling the brown and black particles due to their similarity in appearance to non-plastic items. Microplastic-like particles were divided into foam (lightweight particles with spongy texture), fragments (jagged and irregular shape particles which often have an uneven surface), fibers/filaments (thin, straight and often cylindrical particles), films (thin plane of flimsy particles), or beads (rounded particles)^51^. The selected particles were photographed using a camera apparatus (AxioCam, ERc 5S, Germany). To measure the size of particles, digital images were examined using ImageJ software. Because of the irregularity of most of the isolated particles, sizes of the largest cross-section was measured.

#### Raman spectroscopy

Particles were analyzed over a range of 150 to 3000 cm^−1^ using a Raman spectrometer (Horiba LabRam HR Evolution) equipped with a Single Mode Open Beam Laser Diode (Innovative Photonic Solutions) operating at a wavelength of 785 nm coupled with a charge-coupled device detector (Horiba Synapse). The experimental conditions were adapted as much as possible to limit fluorescence and increase the spectral quality of the measured particles. Another laser line (514 nm) was tested but due to the high level of fluorescence in the visible range, the laser 785 nm was selected, even though some fluorescence was still observed using this wavelength. The acquisition time and number of accumulations were adjusted for each scan such that the detector did not saturate and that the signal to noise was sufficient for performing a library search. A high numerical aperture objective (100X with NA 0.90) was used to increase the signal collection. The confocal hole was partially closed to limit the collection of the fluorescent background. The laser power was set low (below 3 mW) to avoid burning or damaging the material. The low power of the laser did not permit complete efficient bleaching; however, since the acquisition time and the number of accumulations were increased whenever necessary, some photobleaching occurred while accumulating.

Before the library search, to reduce noise and enhance the spectrum quality without losing subtle spectral information, each spectrum passed through a baseline correction and denoising procedure (Labspec 6, Horiba Scientific). Pre-processed spectra were then evaluated and compared to the following spectral libraries: Raman polymers and monomers from Bio-Rad Sadtler and Raman Forensic from Horiba using the KnowItAll software from Bio-Rad. The Correlation algorithm (KnowItAll, Bio-Rad) was used to evaluate each query spectrum to the spectra of the databases. The Hit Quality Index (HQI) was used to rank the results of a spectral search. The HQI, which was scaled between 0 and 1000, indicates how well each spectrum from the database matches the test spectrum. The HQIs > 700 were accepted as evidence of a reliable match between the unknown and the reference spectrum. Furthermore, to investigate the possibility of MP leakage from the packing material to the salts, the chemical composition of the plastic packaging was also determined.

### Contamination prevention

To avoid contamination, cotton lab coat and nitrile gloves were worn at all times. All liquids (deionized water, ethanol) were filtered through 2.7 μm glass fiber filter membranes. The glassware were washed once with dishwashing liquid, then with deionized water, and finally with ethanol. The work surface was thoroughly cleaned with 70% ethanol. Procedures were carried out in a horizontal laminar flow cabinet (Model AHC- 4A1-ESCO) to prevent contamination with airborne MPs. One procedural blank containing only filtered deionized water was tested simultaneously during the extraction procedure and another procedural blank containing only the NaI solution was tested simultaneously during the density separation process, to account for any potential contamination.

## Additional Information

**How to cite this article:** Karami, A. *et al*. The presence of microplastics in commercial salts from different countries. *Sci. Rep.* **7**, 46173; doi: 10.1038/srep46173 (2017).

**Publisher's note:** Springer Nature remains neutral with regard to jurisdictional claims in published maps and institutional affiliations.

## Supplementary Material

Supplementary Information

## Figures and Tables

**Table 1:** Detailed information on the salt samples analyzed in this study.

Country of origin	Brand	Packaging material	Salt type
Australia	A	PE^1^+PET^2^	Sea
	B	PE	Sea
France	C	PP^3^	Sea
	D	PP	Sea
	E	PET	Sea
	F	Glass	Sea
	G	PE+PP	Sea
	H	PET	Sea
Iran	I	PP+Hostasol green	Lake
Japan	J	PE+PET	Sea
Malaysia	K	PP	Sea
	L	PP	Lake
New Zealand	M	PE	Unidentified
Portugal	N	PET	Sea
	O	PP	Sea
	P	Glass	Sea
South Africa	Q	PET	Sea

^1^Polyethylene.

^2^Polyethylene terephthalate.

^3^Polypropylene.
