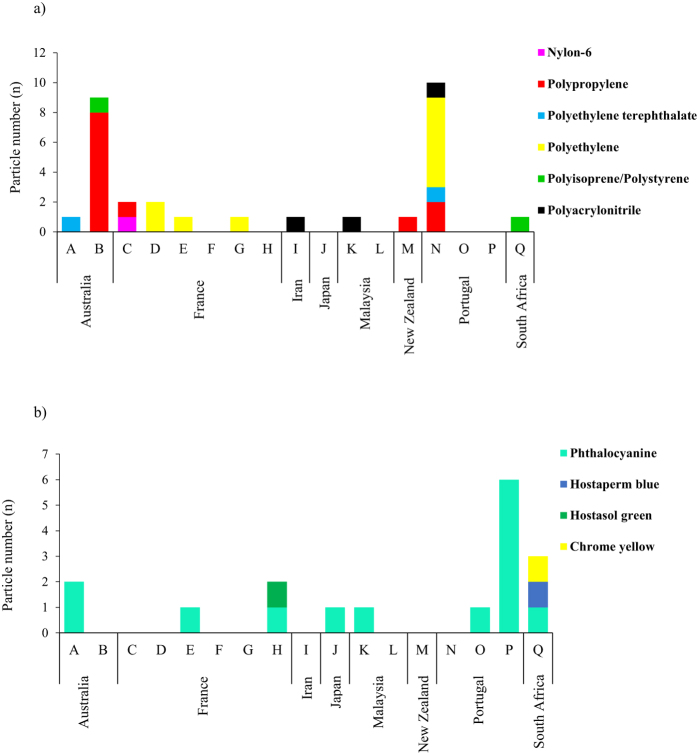# Corrigendum: The presence of microplastics in commercial salts from different countries

**DOI:** 10.1038/srep46838

**Published:** 2017-06-26

**Authors:** Ali Karami, Abolfazl Golieskardi, Cheng Keong Choo, Vincent Larat, Tamara S. Galloway, Babak Salamatinia

Scientific Reports 7: Article number: 46173; 10.1038/srep46173 published online: 04 06 2017; updated: 06 26 2017.

In this Article, there is an error in Figure 3 where panels a and b were inverted. The correct Figure 3 appears below as Figure 1.

## Figures and Tables

**Figure 1:**