# Supplementary material for: The presence of microplastics in commercial salts from different countries
Source: Sci Rep. 2017 Apr 6;7:46173. doi: 10.1038/srep46173 (PMC5382780; doi:10.1038/srep46173)
Supplement: Supplementary Information [file srep46173-s1.pdf]

The presence of microplastics in commercial salts from different countries

Ali Karami*, Abolfazl Golieskardi, Cheng Keong Choo, Vincent Larat, Tamara S. Galloway & Babak Salamatinia

Supplementary Information

Figure 1. Micro-Raman spectra. Spectrum of a: a) blue filament isolated from the salt # Portugal-N (Country of origin: Portugal, brand N) matching with the polyacrylonitrile and Victoria blue spectra, b) pink fragment extracted from the salt # New Zealand-M matching with the polypropylene and Naphthol Red (Pigment red 170) spectra, c) film extracted from the salt # France-E displays the polyethylene and phthalocyanine spectra, d) filament from the salt # Australia-A matching with the polyethylene terephthalate spectrum. Panel e shows a black fragment from the salt # South Africa-Q matching the amorphous carbon spectrum.

a)

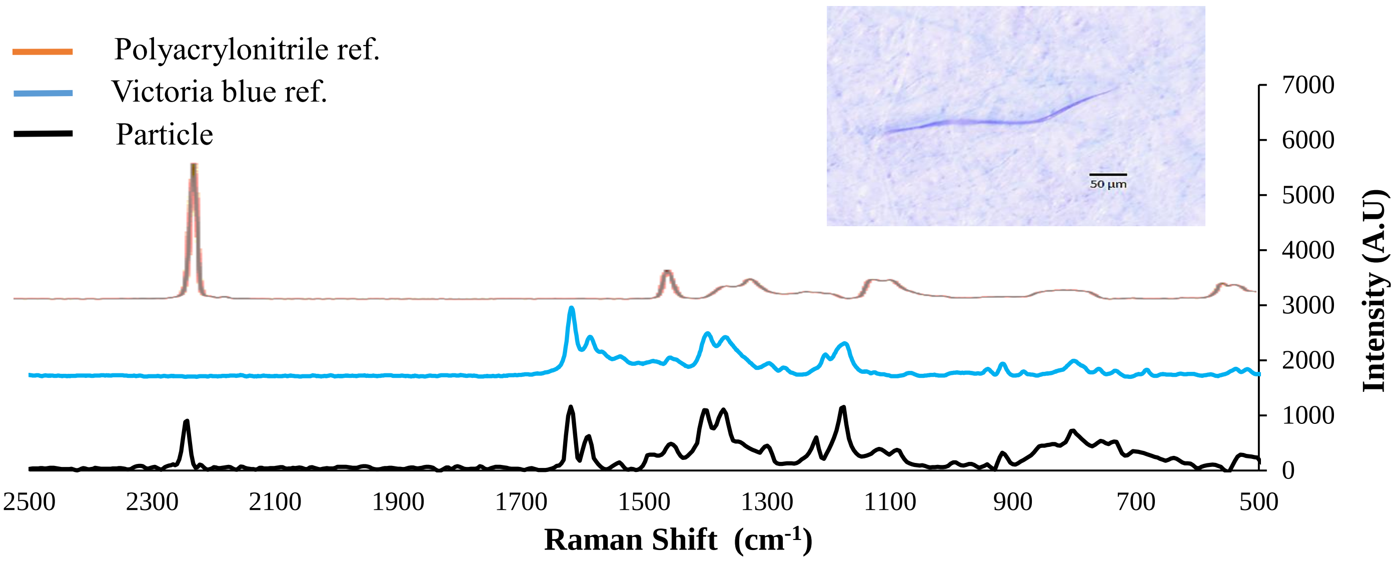
Polyacrylonitrile ref.
Victoria blue ref.
Particle
50 µm
7000
6000
5000
4000
3000
2000
1000
0
Intensity (A.U)
2500
2300
2100
1900
1700
1500
1300
1100
900
700
500
Raman Shift (cm-1)

b)

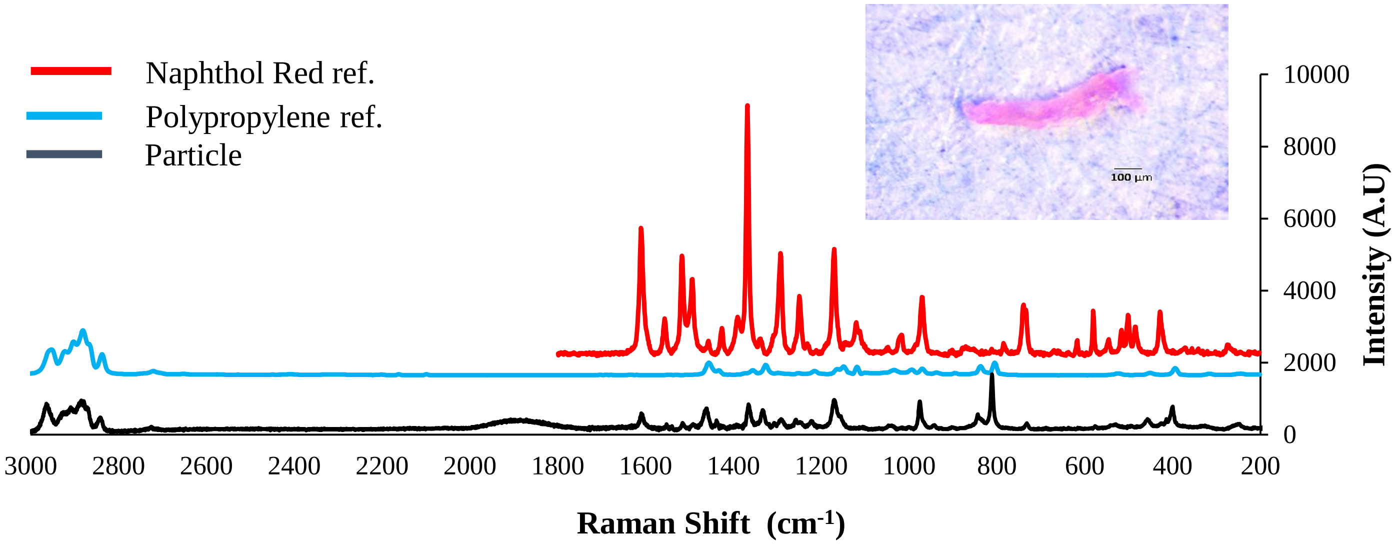
Naphthol Red ref.
Polypropylene ref.
Particle
100 µm
10000
8000
6000
4000
2000
0
Intensity (A.U)
3000
2800
2600
2400
2200
2000
1800
1600
1400
1200
1000
800
600
400
200
Raman Shift (cm-1)

c)

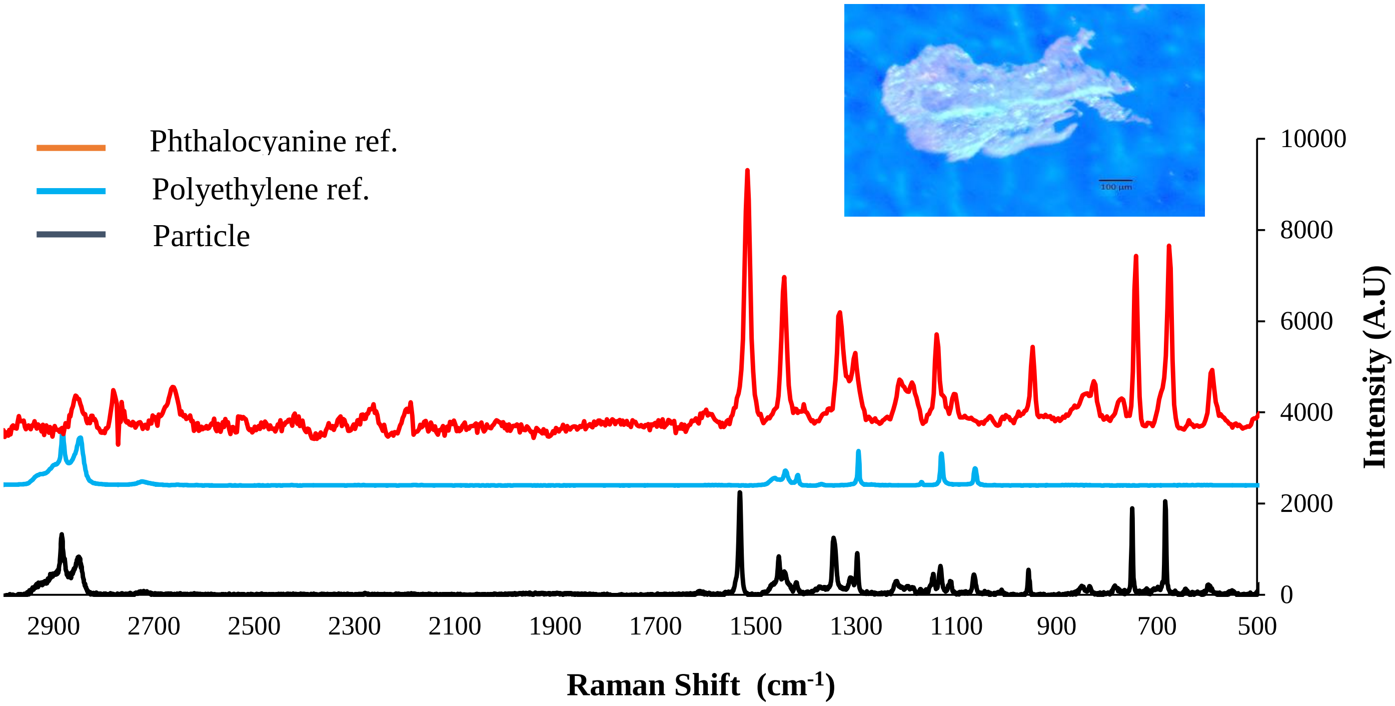

Phthalocyanine ref.
Polyethylene ref.
Particle
10000
8000
6000
4000
2000
0
Intensity (A.U)
2900
2700
2500
2300
2100
1900
1700
1500
1300
1100
900
700
500
Raman Shift (cm-1)

d)

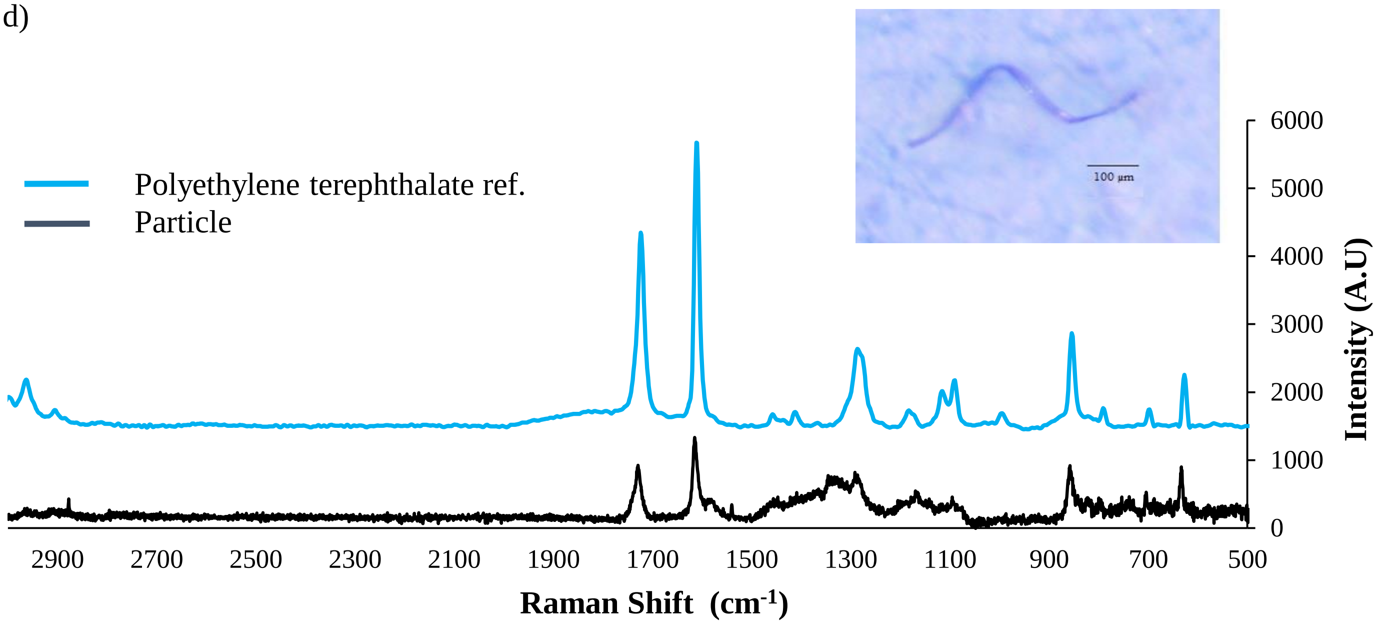

Polyethylene terephthalate ref.
Particle
100 µm
6000
5000
4000
3000
2000
1000
0
Intensity (A.U)
2900
2700
2500
2300
2100
1900
1700
1500
1300
1100
900
700
500
Raman Shift (cm-1)

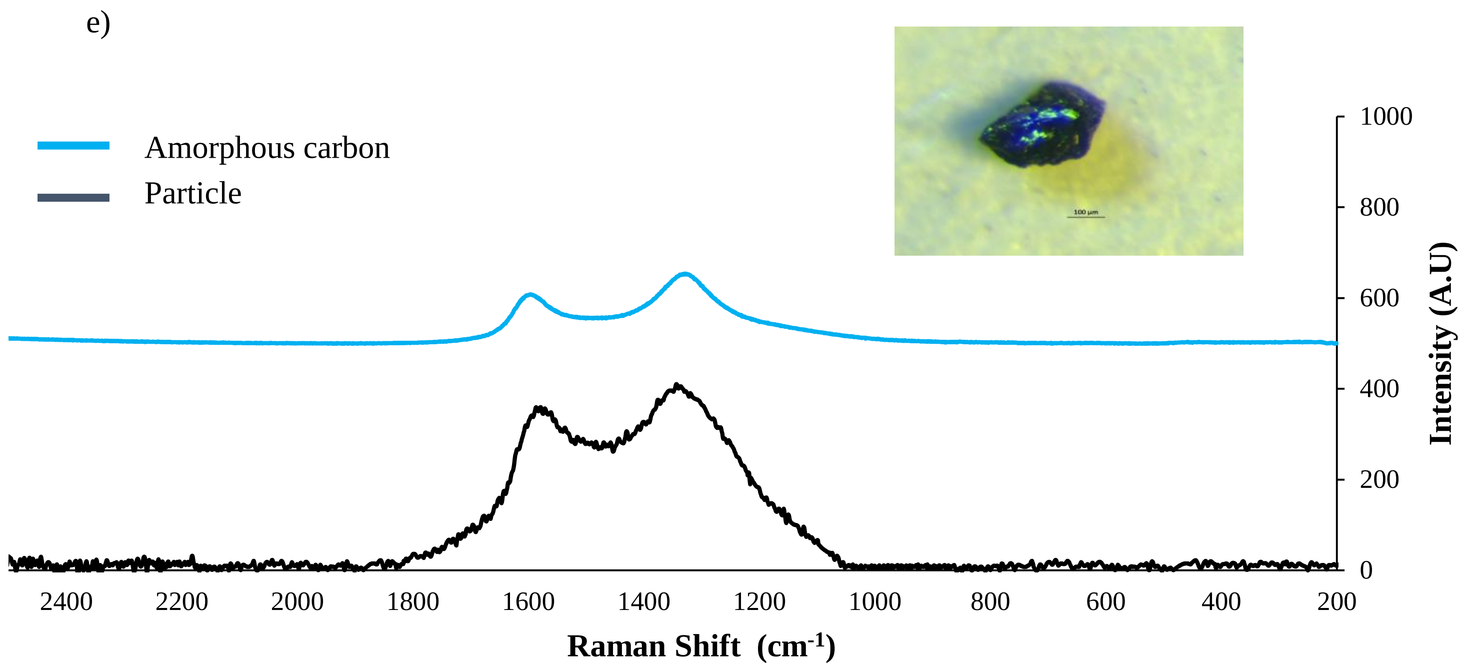
e)
Amorphous carbon
Particle
1000
800
600
400
200
0
Intensity (A.U)
2400
2200
2000
1800
1600
1400
1200
1000
800
600
400
200
Raman Shift (cm-1)
